# Revealing the subtyping of non‐small cell lung cancer based on genomic evolutionary patterns by multi‐region sequencing

**DOI:** 10.1002/cam4.3541

**Published:** 2020-10-20

**Authors:** Gaoming Liao, Xin Liang, Yanyan Ping, Yong Zhang, Jianlong Liao, Yihan Wang, Xiaobo Hou, Zedong Jiang, Xiaoqiu Dong, Chaohan Xu, Yun Xiao

**Affiliations:** ^1^ College of Bioinformatics Science and Technology Harbin Medical University Harbin Heilongjiang China; ^2^ The Fourth Hospital of Harbin Medical University Harbin China

**Keywords:** evolutionary pattern, intra‐tumor heterogeneity, non‐small cell lung cancer, phylogenetic tree, tumor evolution

## Abstract

Accurately classifying patients with non‐small cell lung cancer (NSCLC) from the perspective of tumor evolution has not been systematically studied to date. Here, we reconstructed phylogenetic relationships of somatic mutations in 100 early NSCLC patients (327 lesions) through reanalyzing the TRACERx data. Based on the genomic evolutionary patterns presented on the phylogenetic trees, we grouped NSCLC patients into three evolutionary subtypes. The phylogenetic trees among three subtypes exhibited distinct branching structures, with one subtype representing branched evolution and another reflecting the early accumulation of genomic variation. However, in the evolutionary pattern of the third subtype, some mutations experienced selective sweeps and were gradually replaced by multiple newly formed subclonal populations. The subtype patients with poor prognosis had higher intra‐tumor heterogeneity and subclonal diversity. We combined genomic heterogeneity with clinical phenotypes analysis and found that subclonal expansion results in the progression and deterioration of the tumor. The molecular mechanisms of subtype‐specific Early Driver Feature (EDF) genes differed across the evolutionary subtypes, reflecting the characteristics of the subtype itself. In summary, our study provided new insights on the stratification of NSCLC patients based on genomic evolution that can be valuable for us to understand the development of pulmonary tumor profoundly.

## Graphical abstract

The NSCLC patients were grouped into three subtypes based on the genomic evolutionary patterns. The phylogenetic trees among subtypes exhibited distinct branching structures. The molecular mechanisms of subtype‐specific EDF genes differed across the evolutionary subtypes.

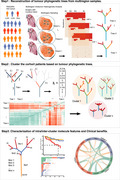

## 1 INTRODUCTION

Non‐Small Cell Lung Cancer (NSCLC) is one of the most common types of lethal cancer, and its pathogenesis is greatly concerned.^1^, ^2^ In recent years, researchers have mapped the complex genomic landscape of NSCLC through large‐scale sequencing studies, which provides an unprecedented reference for diagnosis and treatment.^3^, ^4^, ^5^, ^6^ Intra‐tumor heterogeneity (ITH) may foster tumor adaptation and lead to therapeutic failure through Darwinian selection and has recently been explored widely in cancer based on multi‐region whole‐exome sequencing (WES) data.^7^, ^8^, ^9^, ^10^ Due to the extensive ITH of NSCLC patients, the bottleneck in the treatment emerges, which seriously interferes with the implementation of personalized treatment.^11^, ^12^

Cancer evolution results from the interplay of random events and limits predictability due to stochastic forces, which hinders the selection efficacy of genomic events and leads to complex genomic patterns.^13^, ^14^ Building the tumor phylogenetic relationships can help researchers to depict the genomic evolutionary patterns of tumor patients. Through genetic architecture and evolutionary histories of phylogenetic trees for renal‐cell carcinomas patients, previous studies have revealed branched evolutionary patterns instead of linear model.^7^, ^8^ Also, the researchers observed the parallel evolutionary and convergent evolutionary patterns in patients utilizing the phylogenetic trees and portrayed the polyclonal seeding patterns based on the subclonal structure.^15^, ^16^ Cumulative evidence showed evolutionary potential as a biomarker for both clinical prevention and intervention, such as patient classification, prognosis prediction, and progression surveillance even drug response.^17^, ^18^, ^19^ However, the strategies of personalized medicine based on the evolutionary patterns still have not been well proposed.^6^, ^20^, ^21^

**FIGURE 1:**
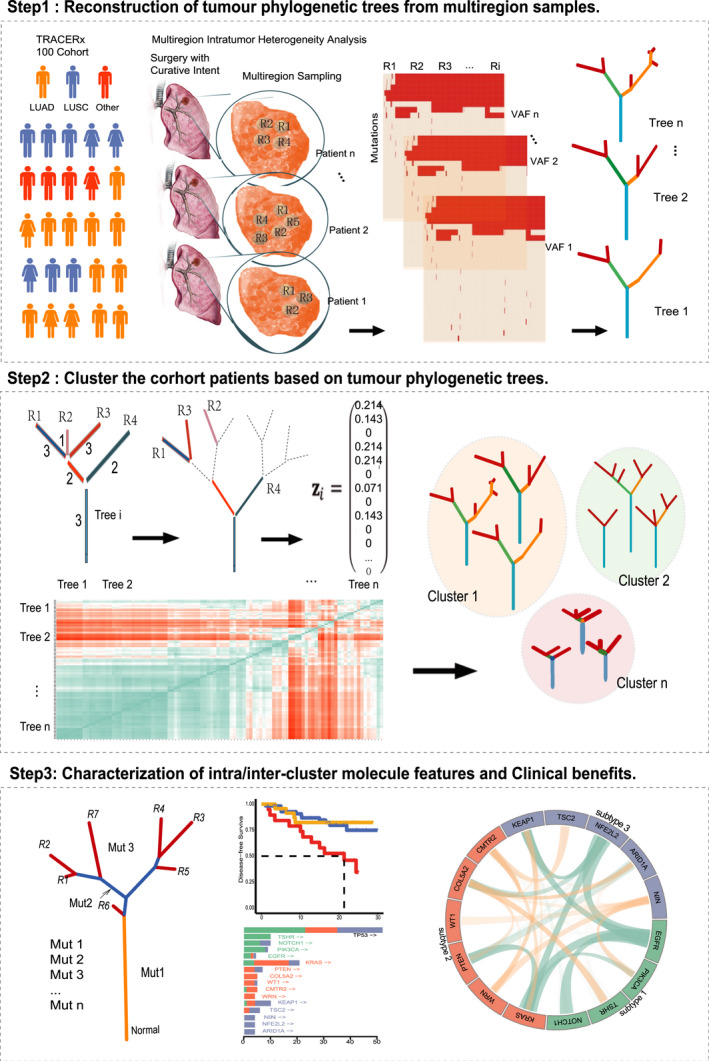
The overview. Step1, The phylogenetic trees of tumor somatic mutations were constructed based on multi‐regional sequencing data from 100 NSCLC patients in the TRACERx cohort. Step2, Clustering the phylogenetic trees and forming evolutionary subtypes. Step3, Characterize the clinical features and molecular mechanisms of genomic variation reflected in subtypes

The Tracking Non‐Small‐Cell Lung Cancer Evolution through Therapy (TRACERx) consortium provides a comprehensive, multi‐centered, and prospective NSCLC patient population.^21^, ^22^ To establish a subtyping model of NSCLC patients from the perspective of genomic evolution, we here reconstructed the phylogenetic trees for 100 patients by reanalyzing the TRACERx data, and classified tumor patients based on the genomic evolutionary pattern presented on the trees (Figure 1). There are significant differences in both evolutionary patterns and survival time across various subtypes. The association between evolutionary subtypes and clinical phenotypes indicates the necessity of analyzing the development of NSCLC from the perspective of tumor genomic evolution.

## 2 MATERIALS AND METHODS

### 2.1 Patients and samples

We obtained the multi‐region sequencing data of 100 NSCLC patients (a total of 327 lesions, including 323 primary lesions and 4 lymph node metastases) without previous therapy from the TRACERx consortium. The data were available from the supplementary material of TRACERx Lung study.^21^ Excision of research tissue from newly exposed tumor surface. At least two regions from each tumor, separated by at least 3 mm, were collected for research purposes. The tumor tissue from each region was used for genomic DNA extraction using a modification of the DNA/RNA AllPrep kit (Qiagen). The single‐nucleotide variants were called in the original study. The driver mutations were identified in the COSMIC cancer gene census (v75) (cancer.sanger.ac.uk), previous large‐scale non‐small cell lung cancer sequencing studies,^3^, ^4^, ^23^ and large‐scale pan‐cancer analyses.^24^ Besides, we obtained mutation data of 1144 TCGA patients (including 660 LUADs and 484 LUSCs) from the Pan‐Lung cohort in cBioPort.^23^

### 2.2 Phylogenetic analysis

All non‐synonymous mutations were used to reconstruct tumor phylogenetic trees. Variant with VAF less than 0.1 were considered as absent. For each NSCLC patient, a tree was rebuilt using the binary distribution of mutations from the region within the tumor. The R Bioconductor package “phangorn” was utilized to perform the Dollo parsimony method^25^ inferring the phylogenetic relationship. The normal sample was designated as the outgroup. For multiple mutations of the same gene, the specific amino acid changes were indicated. Phylogenetic trees were redrawn in Adobe Illustrator with the length of trunks and branches proportional to the number of non‐synonymous mutations.

### 2.3 Mapping the phylogenetic tree

To make the trees comparable, we first encoded the reference tree (a bifurcated tree) for each phylogenetic tree conform to the "maximum depth" of the observed tree.^26^ Then we mapped the phylogenetic trees onto the reference tree according to the edge length beginning with larger, and record it. An example was shown in detail (Figure S1): suppose a tree has a total of seven nodes (including one root node and four leaf nodes) and six edges. The length of the trunk is 3, and the branch lengths of four‐leaf nodes are 3, 1, 3, and 2, respectively, and the length of the intermediate edges is 2. The depth of leaf nodes R1, R2 and R3 are all 3. We added a virtual edge in the middle and changed it to a bifurcated tree. The depth of these three nodes becomes 4. The leaf node R4 has a depth of 2, thus, two virtual edges are added at the end of the node R4 to ensure that all intermediate nodes have two branches. The added virtual edge has a branch length of zero. Then we standardize the length of the edges. The normalized length of the trunk of 0.214 (3/14, the sum of all edge lengths is 14), and the length of the intermediate edges is 0.143 (2/14). From the root node to the leaf node, beginning with larger edge length of subtree, the standardized edges length is recorded using a vector *Zi* = (0.214, 0.143, 0, 0.214, 0.214, 0, 0.071, 0, 0.143, 0,…, 0).

### 2.4 Classification of NSCLC patients

Using the Euclidean distance, we measured the similarity of the evolutionary patterns between the phylogenetic trees in 100 NSCLC patients. The Euclidean distance is calculated as follows:

$$d\left(x_{i},x_{j}\right)=\sqrt{\left(z_{i}‐z_{j}\right)\prime \left(z_{i}‐z_{j}\right)},$$

where *x_i_* is the *i*‐th phylogenetic tree, *z_i_* represents the genomic evolutionary patterns that present in the phylogenetic tree.

Based on the matrix of the evolutionary pattern similarity, the phylogenetic trees were classified by hierarchical clustering. To determine the optimal number of categories for the 100 phylogenetic trees, we calculated the average silhouette coefficient for the number of categories from 1 to 10. The silhouette coefficient is calculated as follows:

$$S_{i}=\frac{b(i)‐a(i)}{\max\{a(i),b(i)\}},$$

where *a*(*i*) represents the average Euclidean distance between the *i*‐th phylogenetic tree and others within the cluster. While *b*(*i*) represents the minimum value of the average Euclidean distance between the *i*‐th tree and trees from other clusters, that is, inter‐class distance. The silhouette coefficient value is consistent with the classification performance of the model.

### 2.5 Calculate branched diversity, ITH index, and CNAS ITH

We obtained non‐synonymous mutations at different locations in the phylogenetic tree, including mutations in alone lesion (Private), mutations shared with some lesions (Shared), and mutations in all lesions (Ubiquitous). Branched diversity was calculated using the number of these three types of mutations, and defined as:

$$\text{Branched diversity}=\frac{\#\text{Private}+\#\text{Shared}}{\#\text{Ubiquitous}},$$

where #Private represents the number of private mutations, #Shared and #Ubiquitous represents the number of shared mutations and ubiquitous mutations, respectively.

We used the clonality of mutations that was determined by observed cancer cell fraction (CCF) to calculate the ITH index and characterize the intra‐tumor heterogeneity for each patient. The ITH index is calculated as follows:

$$\text{ITH index}=\frac{\#\text{Subclonal mutations}}{\#\text{Clonal mutations}},$$

where #Subclonal mutations and #Clonal mutations represent the number of subclonal mutations and clonal mutations, respectively. Mutations were classified as clonal or subclonal based on presence or absence using PyClone.^21^, ^27^

The copy‐number alterations ITH (CNAs ITH) was defined as the numbers of the genome subjected to subclonal CNA (CNA found in some but not all tumor regions) divided by the numbers of the genome subjected to CNA in any region (total CNA).

### 2.6 Calculate the corrected tumor size

For each patient, we approximated the tumor mass as a sphere, and the tumor size after correcting the number of samples was defined as:

$$\text{Corrected TS}=2\cdot \sqrt[{3}]{\frac{{\left(\text{TS}/2\right)}^{3}}{\text{SS}}},$$

where TS is the tumor size and SS is the sample size.

### 2.7 Identify early driver feature (EDF) genes

According to the distribution of mutations on the phylogenetic tree, trunk mutations (Ubiquitous, denoted by A) are generally thought to occur at the earliest stages of tumor genome evolution.^20^, ^28^ The intermediate branch mutations (Shared, indicated by B) are relatively early. The branch mutations (Private, denoted by C) usually tend to occur in the late stages of genome evolution. We identified the Early Driver Feature (EDF) genes according to the following criteria:

1. The priority of driver mutations is determined based on the phylogenetic tree, ubiquitous mutations prior to shared mutations, and shared mutations prior to private mutations.
2. Counts the timing pairs of driver mutations on each phylogenetic tree, including "A‐>B," "A‐>C," and "B‐>C."
3. Requires at least three patients to carry early driver mutations.

### 2.8 Statistical analysis

The Mann–Whitney U test was used to compare continuous variables between the two groups. Survival curves were constructed using the Kaplan–Meier method, and log‐rank tests were used to evaluate the statistical significance of differences. For all statistical analyses, we used R software (version 3.5.2) and considered *p*‐values less than .05 were statistically significant.

## 3 RESULTS

### 3.1 Depiction of intra‐tumor heterogeneity with phylogenetic trees.

**FIGURE 2:**
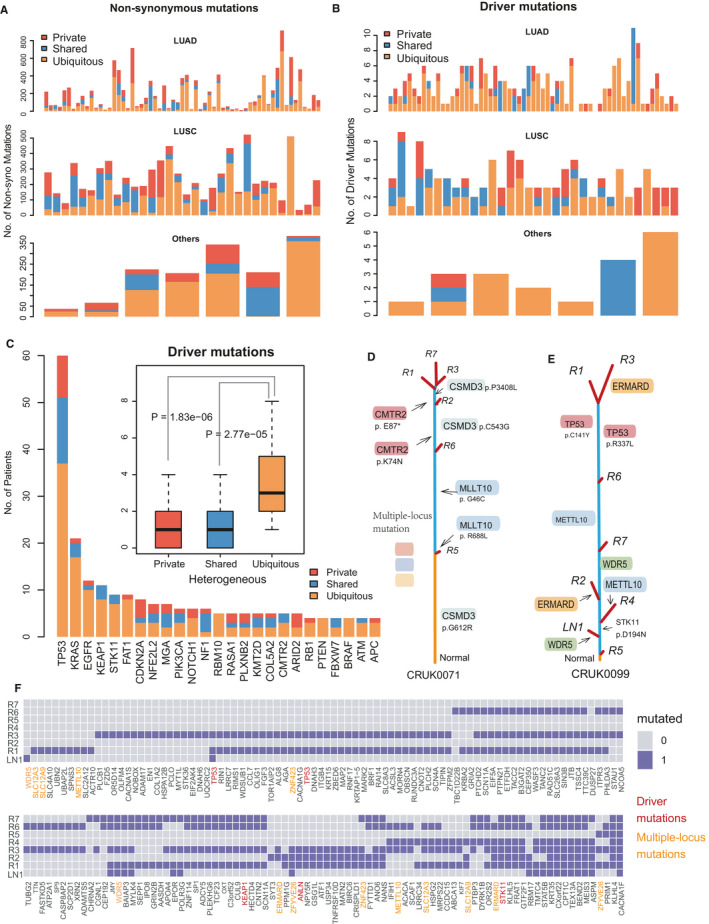
Description of Intra‐tumor heterogeneity and genomic evolutionary diversity. AB, Number of non‐synonymous mutations (A) and driver mutations (B) in locations of the phylogenetic trees across pathological subtype. Yellow (Ubiquitous mutations), blue (Shared mutations), red (Private mutations). C, The number of top 25 driver mutations in different locations of the phylogenetic tree. DE, The phylogenetic tree displays multiple‐locus mutations carried by different lesions of patients CRUK0071 (D) and CRUK0099 (E). Mutations in *METTL10*, *WDR5*, and *ERMARD* present parallel evolution in CRUK0099. F, Binary heat maps show the distribution of multiple‐locus mutations in CRUK0099 patients (CRUK0071 in Figure S4C)

We collected the multi‐region mutation data from 100 NSCLC patients (a total of 327 lesions, including 323 primary lesions and 4 lymph node metastases) without previous therapy^21^ (Figure S2A). The details of mutations identified from the cohort were presented in Figure S2B‐D. The tumor phylogenetic trees were reconstructed for all NSCLC patients using the maximum parsimony method (Figure S3; see Materials and Methods). We found that the mutations in lung squamous cell carcinoma (LUSC) were more likely located in the branch (shared and private mutations) of the phylogenetic trees compared to lung adenocarcinoma (LUAD) (*p* = 0.0017) (Figure 2A). Also, the driver mutations of LUSC patients showed a consistent phenomenon (Figure 2B), which indicated that LUSC had stronger ITH and evolutionary diversity. Furthermore, we found that driver mutations preferred to present in the trunk in most NSCLC patients (Figure 2C). The 25 genes with the highest variant frequency showed that the driver mutations were more likely to be ubiquitous mutations (*p* = 2.77e‐05 and *p* = 1.83e‐06 compared to shared and private mutations, respectively). For example, the ubiquitous mutations of *KRAS* and *EGFR* presented in 81% (17/21) and 83% (10/12) patients, respectively. These results reflected the early potential of driver mutations during tumor evolution.^21^, ^29^, ^30^

In the phylogenetic trees, we observed that different branches carried multiple‐locus mutations of the same gene (Figure 2D,E). For instance, patient CRUK0071 carried driver mutations of *CSMD3* at three amino acid positions, including the site p.G612R was located in the trunk of the tree, while both sites p.C543G and p.P3408L were located in shared branches (Figure 2D). The distribution of multiple‐locus mutations and driver mutations in different lesions of CRUK0099 are shown in Figure 2E,F. Interestingly, parallel evolutionary patterns were exhibited in the phylogenetic trees, including mutations in *MLLT10*, *WDR5*, and *ERMARD* (Figure 2D,E), which indicated the diversity of tumor genome evolution. In summary, the phylogenetic relationships built with multi‐region data can help us to characterize the ITH of tumor tissue and spatial diversity of genomic variation.

### 3.2 Classification of tumor patients based on evolutionary patterns.

**FIGURE 3:**
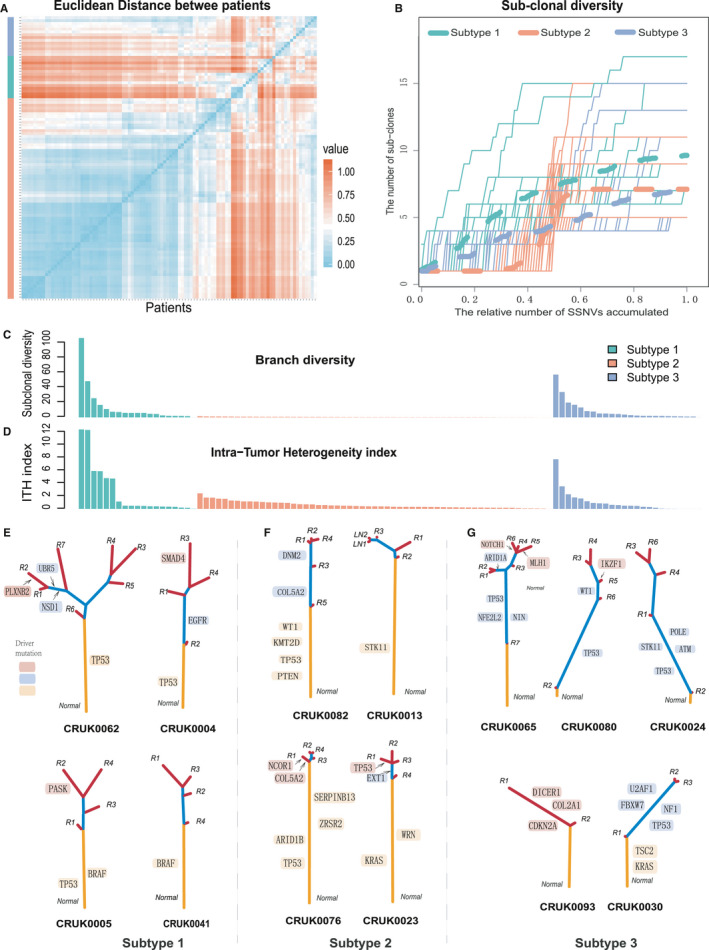
Subtyping NSCLC patients using genomic evolutionary patterns. A, The hierarchical clustering was used to cluster the genomic evolutionary patterns presented in phylogenetic trees. The greener the color, the greater the similarity between the trees. B, The accumulated mutations were used to characterize the patient's sub‐clonal diversity. *X*‐axis, the number of accumulated mutations was subdivided into steps of 0.01 and normalized to 0‐1. *Y*‐axis, The number of subclonal mutations that measured by the distance from the root node of the phylogenetic tree to all other nodes is less than the fraction of accumulated mutations. The larger the area under the curve, the stronger the subclonal diversity. C, Characterization of branched diversity in patients using non‐synonymous mutations at different locations of the phylogenetic trees. D, Reveal the patient's Intra‐tumor heterogeneity index based on the clonality of the mutations. E‐G, The phylogenetic trees from subtype 1 (E), subtype 2 (F), and subtype 3 (G). Branch lengths are proportional to the number of non‐synonymous mutations in each tree

The clustering method for phylogenetic trees based on the evolutionary patterns can help to detect the phenotype‐related subgroups.^26^ In our study, we explored evolutionary subtypes of 100 NSCLC patients according to evolutionary patterns of phylogenetic trees (Figure 1). To make the phylogenetic trees comparable, we mapped each tree onto a reference tree (see Materials and Methods). The Euclidean distance was used to measure the similarity of evolutionary patterns between phylogenetic trees (Figure 3A; Table S1). At the best clustering effect, 100 NSCLC phylogenetic trees were clustered into three groups (called evolutionary subtypes; the number of patients was 19, 57, and 24, respectively) using the hierarchical clustering method (Figure 3A, Figure S4A; see Materials and Methods). The similarity of evolutionary patterns across the three subtypes is various (Figure 3A). For instance, among the three evolutionary subtypes, the phylogenetic trees in subtype 2 exhibited the smallest Euclidean distance. Using singular value decomposition to display all phylogenetic trees in two‐dimensional Euclidean space, we found that the trees within subtype 2 were the most compact (Figure S4B).

The patients within subtype 1 exhibited higher subclonal diversity mediated through cumulative mutations in the phylogenetic trees, which reflects higher ITH^26^, ^31^ (Figure 3B). To further characterize ITH across evolutionary subtypes, we calculated branched diversity using non‐synonymous mutations in phylogenetic trees (see Materials and Methods). We found the patients in both subtype 1 (median = 5.85) and subtype 3 (median = 3.31), showed significantly higher branched diversity than subtype 2 (median = 0.30) (*p* = 3.34e‐09 and 9.93e‐11, respectively) (Figure 3C). The branched diversity of subtype 1 tended to be higher than subtype 3 (*p* = 0.09). Similarly, the patients within subtype 1 exhibited the highest ITH index (see Materials and Methods) compared with the other two subtypes (*p* = 0.017 and 0.048, respectively) (Figure 3D). Interestingly, as with mutational heterogeneity, the CNAs ITH also showed the consistent correlations with evolutionary subtypes (Figure S5A). These results indicated that patients with different ITHs allowed us to classify tumor patients based on genomic evolutionary patterns.

The phylogenetic trees of three evolutionary subtypes presented different evolutionary branched structures (Figure 3E‐G). The first subtype consisted of patients with branches of the phylogenetic trees which were similar to the length of trunks and exhibited branched evolutionary pattern.^32^ We pinpointed driver mutations on the trees and found them evenly distributed in trunks and branches. For instance, in patient CRUK0062, the mutation in *TP53* occurred on the trunk, while in *NSD1* and *UBR5* were shared by branches, and in *PLXNB2* was detected only on the private branch (Figure 3E). In this subtype patients, we found that the driver mutation of *TP53* was frequently identified on the trunks, indicating the mutation were acquired relatively early during the evolution of these tumors. This evolutionary pattern showed that multiple clonal lineages acquired sufficient evolutionary fitness during tumor progression and cause expansion. Subsequently, new subclonal lineages formed, in consequence of the positive selection of driver mutations,^33^ which can increase the propensity for drug resistance and will cause poorer clinical outcomes for this type of patients.^11^ In contrast, the trees of the second subtype patients presented long trunks and short branches (Figure 3F). And we found that most driver mutations, such as mutations in *PTEN*, *WT1*, *KRAS*, and *STK11*, etc., were located in the trunks of the phylogenetic trees. The branched structure of the trees suggested that the tumor genomic variation of this type of patient was in the early stage of accumulation, and the subclonal expansion occurs in the late tumor progression.^20^ This pattern would be connected with the improved clinical risk profiles.^11^

However, in the phylogenetic trees of the third subtype patients, the driver mutations were mainly shared by the partial lesions (Figure 3G). For example, in patient CRUK0065, the driver mutations in *TP53*, *NFE2L2*, *ARID1A*, and *NIN* were distributed on the shared branches of the phylogenetic tree. And the length of the trunk was shorter compared to the branches. Further, the evolutionary branch length of most phylogenetic trees was asymmetrical (Figure 3G). The length of the branch shared by R3‐R6 was longer than the branch shared by R1/R2 in CRUK0065, the branch shared by R2/R3 was longer than the R1 branch in CRUK0030. In particular, in patients CRUK0080 and CRUK0024, a small number of mutations were detected in the R2 lesion, and were gradually replaced by newly formed mutations that were distributed in multiple branches of the evolutionary tree. This evolutionary pattern suggested that some mutations cannot afford a fitness advantage to allow for additional driver events, which caused a clonal sweep during tumorigenesis. Unlike linear model,^33^ multiple subclonal lineages formed in this pattern during tumor progression, which was consistent with higher ITH.

### 3.3 Tumor genomic evolution reflects clinical phenotypes and outcomes

**FIGURE 4:**
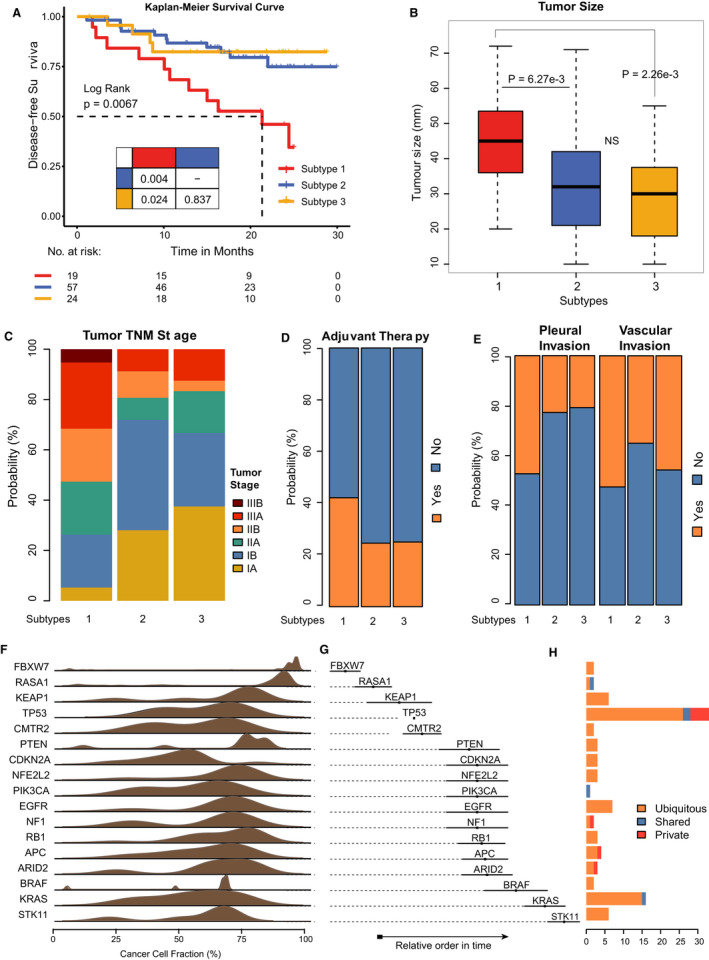
Characterization of clinical phenotypes across evolutionary subtypes. A, The Kaplan–Meier curves regarding disease‐free survival for respective evolutionary subtypes. B‐E, Relationship between evolutionary subtypes and tumor size (B), TNM stage (C), adjuvant therapy (D), and invasion status (E). F, The density distribution of driver‐mutation CCFs in subtype 2 patients. G, The temporal order of driver mutations in the phylogenetic trees of subtype 2. G, Sample coverage of the driver mutations in subtype 2 based on the location on the phylogenetic tree

Intra‐tumor heterogeneity allows tumors to grow and develop along various evolutionary trajectories, which result in diverse clinical phenotypes.^17^ We wanted to know whether evolutionary subtypes of NSCLC patients could reflect clinical phenotypes. Previous researches have shown that evolutionary patterns determined by subclonal architecture cause heterogeneous clinical outcome.^34^, ^35^ In our study, we found that patients across evolutionary subtypes presented significant differences in disease‐free survival (*p* = 0.0067) (Figure 4A). In particular, patients within subtype 1 exhibited significantly worse survival compared to the other two subtypes (*p* = 0.004 and 0.024, respectively) (Figure 4A). We have illustrated that subtype 1 had the strongest ITH and subclonal diversity (Figure 3B‐D). However, there was no significant difference in survival time between subtype 2 and subtype 3, which was consistent with their likeness in ITH (*p* = 0.837). This result indicated that the genomic evolutionary patterns presented in the phylogenetic tree could reflect the patient's survival.

Furthermore, the analysis showed that the patients in subtype 1 had significantly larger tumor size compared to the other two evolutionary subtypes (*p* = 6.27e‐3 and 2.26e‐3, respectively) (Figure 4B). Similar to the survival time, subtype 2 and subtype 3 did not show significant differences in tumor size. After correcting the number of samples, consistent results were presented (Figure S5B). Also, the patients in subtype 1 tended to have a higher proportion of high‐tumor stage (stage IIIA and IIIB, accounting for 31%), and lower proportion of low‐tumor stage (stage IA and IB, accounted for only 26%) (Figure 4C), a higher proportion of underwent adjuvant treatment (42.1%) (Figure 4D), and with more likeliness to have pleural invasion (47.4%) and vascular invasion (52.6%) (Figure 4E). The results of evolutionary subtypes and clinical phenotypic analysis indicated that a large number of subclonal expansions, which resulted in tumor tissue growth, could lead the tumor cells to invade other tissues (including vascular invasion and pleural invasion), and in turn caused the progression and deterioration of the disease and higher stage. Even more, the adjuvant treatment caused patients to form new subclonal populations, further enhancing the patient's ITH, and ultimately lead to poorer survival^11^.

### 3.4 Mutational spatio‐temporality reveals evolutionary subtype‐specific early driving genes

Genome variation events have temporal characteristics during tumor evolution.^36^ The temporal order of genomic changes reveals the phenotypic features of evolutionary subtypes and serves as potential biomarkers to guide intervention and surveillance.^17^ In this study, we evaluated the global trend in temporal order for driver mutations in NSCLC, by comparing the CCFs of coexisting mutations, which were characterized by their positional distribution on the phylogenetic trees based on the Bradley‐Terry model.^37^ By showing the relatively earlier evolutionary timing in subtype 1, driver mutations such as *PIK3CA*,*EGFR*, and *TP53* presented larger CCFs (Figure S6A,B). Previous studies have shown that driver mutations in *EGFR* and *TP53* played a crucial role in the early stages of lung cancer development.^38^ For subtype 2, however, we found that both early and late driver mutations belonged to ubiquitous events (85/98), such as *FBXW7*,*CMTR2*, and *STK11* (Figure 4F‐H). Zhang et al. found that most of the known oncogene mutations occurred in the trunks of the phylogenetic trees, indicating that these mutations were at the early stages of genomic variation in lung cancer evolution.^20^ This result explained that patients within subtype 2 had the strongest homogeneity (Figure 3C,D). In subtype 3, the driver mutations of *NFE2L2* and *TP53* showed a larger CCFs (Figure S6C,D).

To reveal the functions and properties of early driver mutations in detail, we identified early driver genes (Early Driver Feature genes, called EDF genes below) in different patient sets, which exhibited early features on phylogenetic trees based on the temporality of mutations (see Materials and Methods). First, we explored the mutation distribution status of EDF genes in adenocarcinoma and squamous cell carcinoma, separately. By analyzing the number of patients covered, we found that the EDF genes showed the correlations with the specific histology subtype. For instance, all the driver mutations of *KRAS* occurred in adenocarcinoma patients (21/61), while not detected in squamous cell carcinoma patients (Figure S5C). All the driver mutations of *PTEN* occurred just in squamous cell carcinoma patients (7/32). These results were consistent with the study of TCGA Pan‐Lung cohort^23^ (Figure S7A). The EDF genes specific for the histology subtype also include *TSHR*, *KEAP1*, and *EGFR* in adenocarcinoma, and *NOTCH1*, *PIK3CA* in squamous cell carcinoma (Figure S5C, Figure S7A).

**FIGURE 5:**
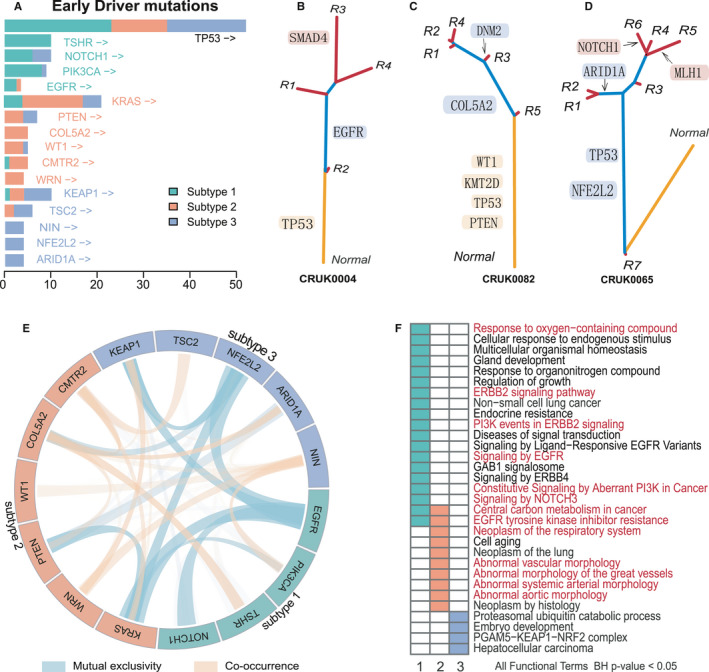
Identification and molecular mechanism of evolutionary‐specific EDF genes. A, Tumor lesions coverage of EDF genes across evolutionary subtypes. The colors corresponding to different subtypes have been displayed. B‐D, Partial EDF genes mutations located in the phylogenetic tree within subtype 1 (B), subtype 2 (C), and subtype 3 (D) patients, respectively. E, The mutual exclusivity and co‐occurrence of EDF genes were inferred based on the Fisher test. Light green indicates mutual exclusivity, orange indicates co‐occurrence. The thickness of the line represents the log odds ratio (LR) and the transparency represents the significance level (*p* < 0.05 were shown). F, Functional enrichment analysis of EDF genes using g:Profiler requires a significance level (Benjamini Hochberg adjusted) of 0.05 or less. The red color indicates the representative functions mentioned in the text

Interestingly, whether in adenocarcinoma or squamous cell carcinoma, we found that the mutation of these EDF genes showed significant aggregation in the specific evolutionary subtype. For example, all the mutations of both *TSHR* (10/10) and *PIK3CA* (7/7) occurred in evolutionary subtype 1 (Figure S5C). The mutations of *WRN* (4/4) and *COL5A2* (4/4) were only occurred in subtype 2, and the mutations of *KEAP1* (7/10), *NFE2L2* (4/4), and *ARID1A* (4/4) were almost only in subtype 3 (Figure S5C). Considering the impact of the sample size, furthermore, we want to know whether these EDF genes present evolutionary subtype specificity in all NSCLC patients. Indeed, the analysis results did show a consistent phenomenon. For instance, in all NSCLC patients, the mutation of EDF gene *PIK3CA* was an early evolutionary event in 89% (8/9) patients within subtype 1 (Figure 5A), which was consistent with the evaluation of the temporal order (Figure S6A,B). While *TSHR* showed early driver mutations in all subtype 1 patients (10/10). Similarly, the driver mutation of *COL5A2* was an early event in 100% (5/5) phylogenetic trees in subtype 2 (Figure 5A). The phylogenetic trees showed the distribution of some EDF genes mutation in each evolutionary subtype (Figure 5B‐D). In summary, by the temporal order of the driver mutations in the phylogenetic tree, we identified evolutionary subtype‐specific EDF genes, including *PIK3CA*, *NOTCH1*, *EGFR*, and *TSHR* in subtype 1 patients; *KRAS*, *PTEN*, *COL5A2*, *CMTR2*, *WT1*, and *WRN* in subtype 2; as well as *NFE2L2*, *KEAP1*, *NIN*, *ARID1A*, and *TSC2* in subtype 3 (Figure 5A; Table S2).

### 3.5 Functional analysis of early driver genes portrays the molecular mechanisms of evolutionary subtypes

To characterize the molecular mechanism of the EDF genes, we used TCGA Pan‐Lung cohort^23^ for subsequent analysis. As a result, we found that these EDF genes presented mutual exclusivity of genomic variation among subtypes (Figure S7A). Thus, we inferred the mutual exclusivity and co‐occurrence relationships between all EDF gene pairs. Indeed, the results showed that EDF genes among subtypes significantly were mutually exclusive, such as, subtype 1 *EGFR* and subtype 2 *KRAS* (LR < −3, *p*‐value < 0.001; Fisher test), as well as *EGFR* and subtype 3 *NFE2L2* (LR < −3, *p*‐value < 0.001), *EGFR* and subtype 3 *KEAP1* (LR = −1.6, *p*‐value < 0.001) (Figure 5E, Table S3). Also, *KRAS* and *NFE2L2* showed significant mutual exclusivity (LR = −1.6, *p*‐value < 0.001). Researches have shown that gain‐of‐function mutations of *EGFR*, *KRAS*, *NFE2L2*, and loss‐of‐function mutations of *KEAP1*, could produce a convergent activation of *NFE2L2* ^39^, ^40^, ^41^ (Figure S7B). While *NFE2L2* has been proved to increase the oxidoreductase activity, to mediate mitochondrial synthesis and energy metabolism, and provide the raw material for genome replication.^42^ This indicated that the mutation of these EDF genes among the subtypes could activate *NFE2L2* in different ways.

Particularly, EDF genes within subtype 2 were more likely to exhibit co‐occurrence of genomic variation, such as *CMTR2* and *COL5A2* (LR = 1.07, *p*‐value = 0.01), *CMTR2* and *KRAS* (LR = 0.72, *p*‐value = 0.03), as well as *COL5A2* and *PTEN* (LR = 0.72, *p*‐value = 0.04) (Figure 5E, Table S3). By functional enrichment analysis of EDF genes in this subtype, we observed that these genes were enriched in pathways associated with abnormal vascular morphology (Figure 5F). A previous study has shown that tumor cell growth critically depended on the formation of abnormal blood vessels to supply nutrition and oxygen.^43^ By cancer hallmarks associated gene sets,^44^ we found that *PTEN* participated in the apical junction complex, abnormalities in which can cause a decrease in adhesion between cells. While *COL5A2* was involved in the "epithelial‐mesenchymal transition" related hallmark, which promoted tumor cell invasion and metastasis. Furthermore, *COL5A2*, *PTEN*, and *KRAS* were enriched in the function like “negative regulation cell differentiation” (data not shown). This result indicated that EDF genes in subtype 2 promoted the development of cancer by obtaining cell stemness, which was consistent with the early accumulation of mutations in this subtype (Figure 3F, Figure 4H).

Further, we found that the EDF genes were enriched in respiratory‐related functions in subtype 1 and subtype 2, such as “Response to oxygen‐containing compound” and “Neoplasm of the respiratory system,” respectively (Figure 5F). Both “Central carbon metabolism in cancer” and “EGFR tyrosine kinase inhibitor resistance” were enriched by the EDF genes of these two subtypes, which may suggest that the early driver genes causing different tumor genomic evolutionary patterns have driven the same functions. Besides, the EDF genes within subtype 1 were enriched in functions of cell growth, such as "ERBB2 signaling pathway" and "Constitutive Signaling by Aberrant PI3K in Cancer" (Figure 5F), which could affect tumor cell growth, proliferation, and migration by perturbing the PI3K/AKT/mTOR signaling pathway.^45^, ^46^, ^47^ Taken together, early driving events among evolutionary subtypes determined that genomic variation could perturb the same or subtype‐specific functions in multiple ways, drove tumorigenesis and progression in various forms, and ultimately led to diverse evolutionary patterns in cancer patients.

## 4 DISCUSSION

Tumor genome evolution has molecular diversity and heterogeneity, which presents the temporal order of genomic variation events during tumor progression.^17^, ^48^ Large number sequencing analyses of NSCLCs have identified extensive genetic heterogeneity within tumor, which can contribute to treatment failure and drug resistance.^6^, ^7^, ^9^ Intra‐tumor heterogeneity may have an important impact on personalized medicine approaches that generally rely on a single‐tumor biopsy samples to portray tumor mutations. Studies have provided evidence of intra‐tumoral heterogeneity at nucleotide resolution by comparing mutation profiles in multi‐regions of the same tumor.^15^, ^18^ Intra‐tumor heterogeneity appears in diverse genomic evolution patterns on the phylogenetic tree.^11^

The researchers tracked the genomic evolution across multiple cancer types and revealed different evolutionary patterns.^15^, ^32^ Gerlinger et al. portrayed the structure and evolutionary history of the genome, revealing that patients presented a branched evolutionary pattern rather than linear evolution, and showed a parallel evolution of driver mutations.^8^ Similar evolutionary patterns of a patient subtype may reflect the clinical implication, including classification, prognosis, and therapy. The branched evolution and linear evolution had different responses to standard treatment in chronic lymphocytic leukemia patients.^49^, ^50^ Karlsson et al. identified four patient subtypes with distinct evolutionary trajectories based on the dynamic clonal architecture in childhood cancer, which was associated with different mutation processes and survival.^19^ A study on 101 patients with clear‐cell renal cell carcinoma proposed a patient stratification strategy based on evolutionary routes and revealed evolutionary subtypes correlated with diverse clinical phenotypes and outcomes.^17^ However, the characterization of genomic patterns from the perspective of tumor evolution is not comprehensive, and strategies for using genomic evolution patterns for personalized treatment have not been well proposed. In this study, we used the multi‐region WES data from the TRACERx NSCLC cohort to build the phylogenetic relationship for 100 patients. The genomic evolution patterns presented by the phylogenetic trees were used to establish the subtyping system for NSCLC patients.

The first subtype was characterized by high subclonal diversity and branched diversity, and presented a branched evolutionary pattern, suggesting multiple clone lineages diverged from a common ancestor and had high fitness with a selected sweep. They had the shortest disease‐free survival time and had the largest tumor size, more likely to be in higher stages, which were consistent with a selected clonal sweep and accelerated tumor growth, due to the presence of additional driver events on the evolutionary branch.^17^ The second subtype showed minimal ITH, and their phylogenetic trees exhibited a long trunk and short branches. Conform to the branching structure of "Palm tree–like" tumors,^11^ the patients in this pattern carry more ubiquitous mutations than heterogeneous mutations and present better survival status. Moreover, the driver mutations were more likely to occur on the trunk of the phylogenetic tree, indicating the early stage of tumor evolution in this subtype. These subtype‐specific EDF genes exhibited co‐occurrence in a population of patients, which was inseparable from the lower ITH. Meanwhile, one evolutionary pattern in the phylogenetic tree was represented by a short trunk and a long intermediate branch that carried most of the driver mutations. Unlike the linear model, an evolutionary branch contains a very small number of mutations and was gradually replaced by multiple newly formed branching structures during tumor progression.

Next, we explored the correlations between evolutionary subtypes and the clinical phenotypes and outcomes of NSCLC patients, and found that the patients with the worst prognostic efficacy showed stronger intra‐tumor heterogeneity and subclonal diversity. Combining the location distribution of mutations in the phylogenetic tree, we found that the driver mutations in different subtypes present diverse evolutionary timing relationships. We finally used early driver mutations to reveal early driver feature genes that reflect subtype specificity. These early driver genes exhibited mutual exclusion among subtypes, and also showed subtype specificity in molecular mechanisms for tumorigenesis. Ultimately, we expect that the application of our results to individualized treatment will be helpful for the development of personalized medicine from the perspective of tumor evolution.

## CONFLICT OF INTEREST

The authors have no conflict of interest.

## AUTHOR CONTRIBUTIONS

Yun Xiao, Chaohan Xu, and Xiaoqiu Dong provided scientific ideas and designed the study. Gaoming Liao and Xin Liang performed all data analysis, designed the figures, and drafted the manuscript. Yanyan Ping, Yong Zhang, Jianlong Liao, Yihan Wang, Xiaobo Hou, and Zedong Jiang performed preliminary data processing and modified the manuscript. All authors read and approved the final manuscript.

## Supporting information

Fig S1

Fig S2

Fig S3

Fig S4

Fig S5

Fig S6

Fig S7

Table S1

Table S2

Table S3

## Data Availability

The authors declare that all data supporting the findings of this study are available within the article and its Supplementary Information files or are available from the corresponding author upon reasonable request.
