# Supplementary figures and images for: Revealing the subtyping of non‐small cell lung cancer based on genomic evolutionary patterns by multi‐region sequencing
Source: Cancer Med. 2020 Oct 20;9(24):9485–98. doi: 10.1002/cam4.3541 (PMC7774747; doi:10.1002/cam4.3541)

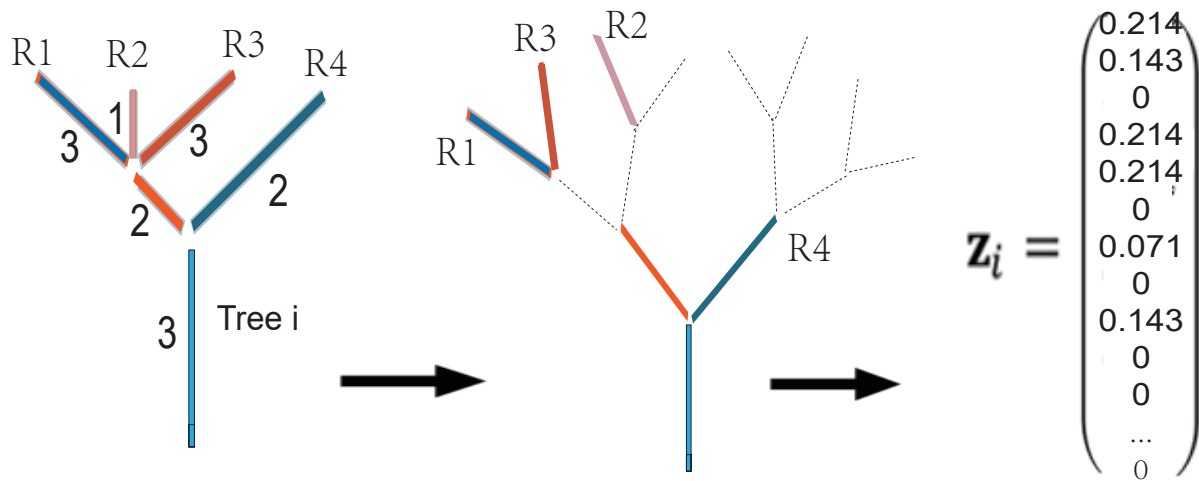

Figure S1. Recording and mapping the phylogenetic trees.

Supplement: Supplementary file 1 — Fig S1 [file CAM4-9-9485-s001.pdf]

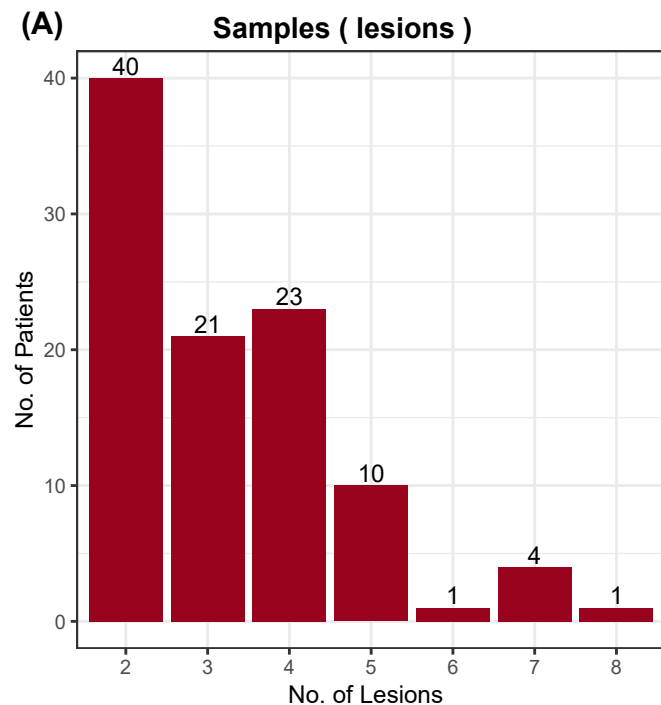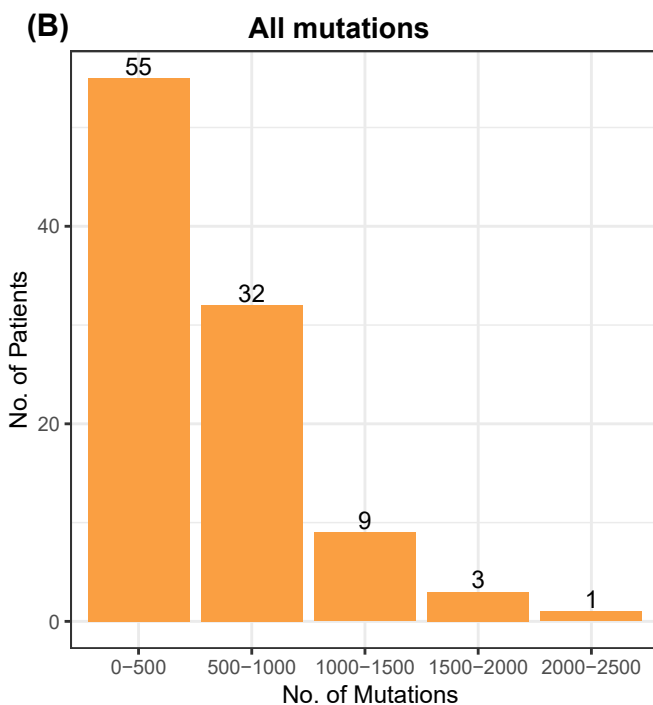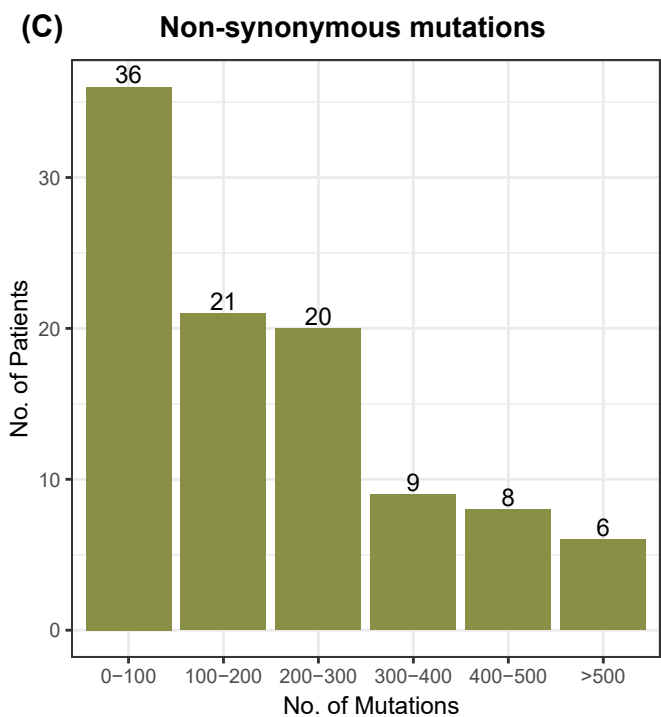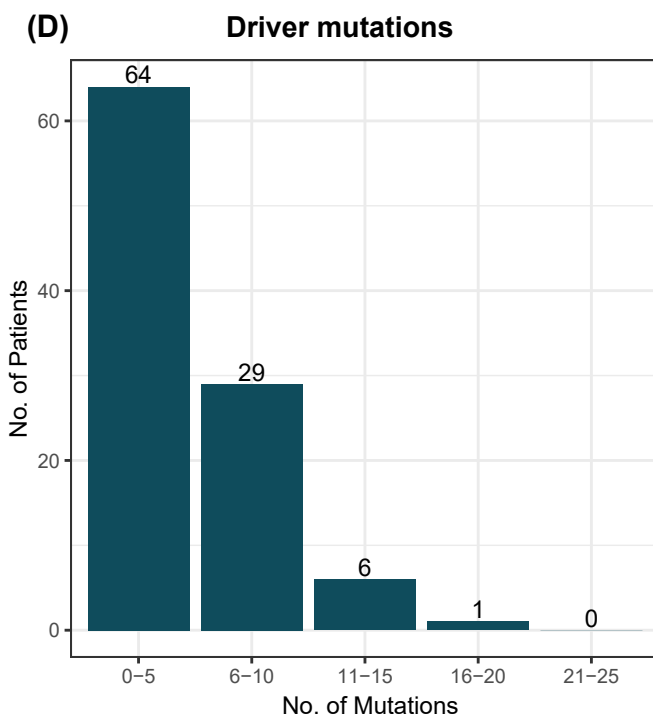

Figure S2. Summary of the number of mutations for 100 NSCLC patients.

Supplement: Supplementary file 2 — Fig S2 [file CAM4-9-9485-s002.pdf]

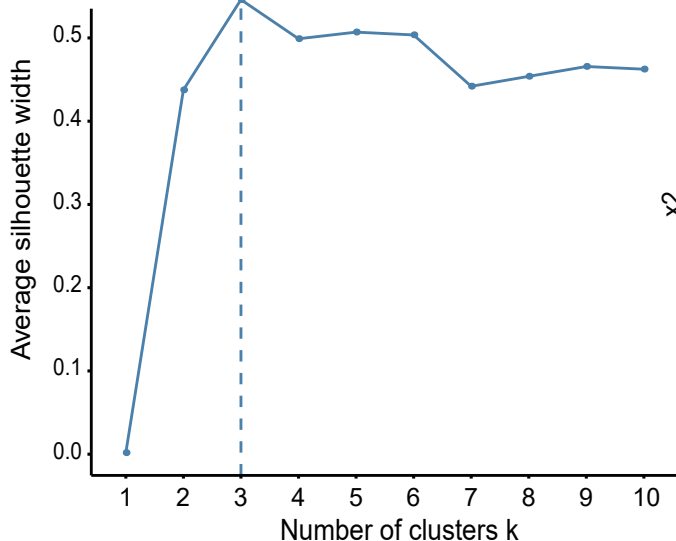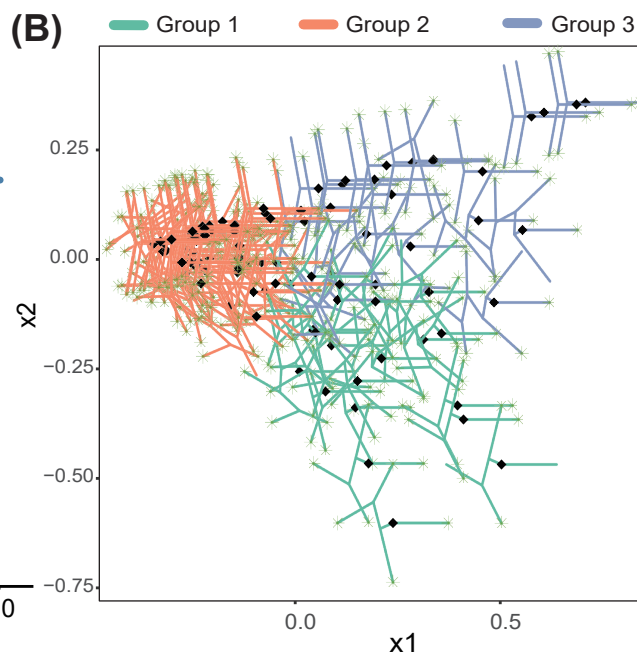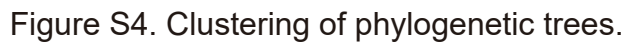

Supplement: Supplementary file 4 — Fig S4 [file CAM4-9-9485-s004.pdf]

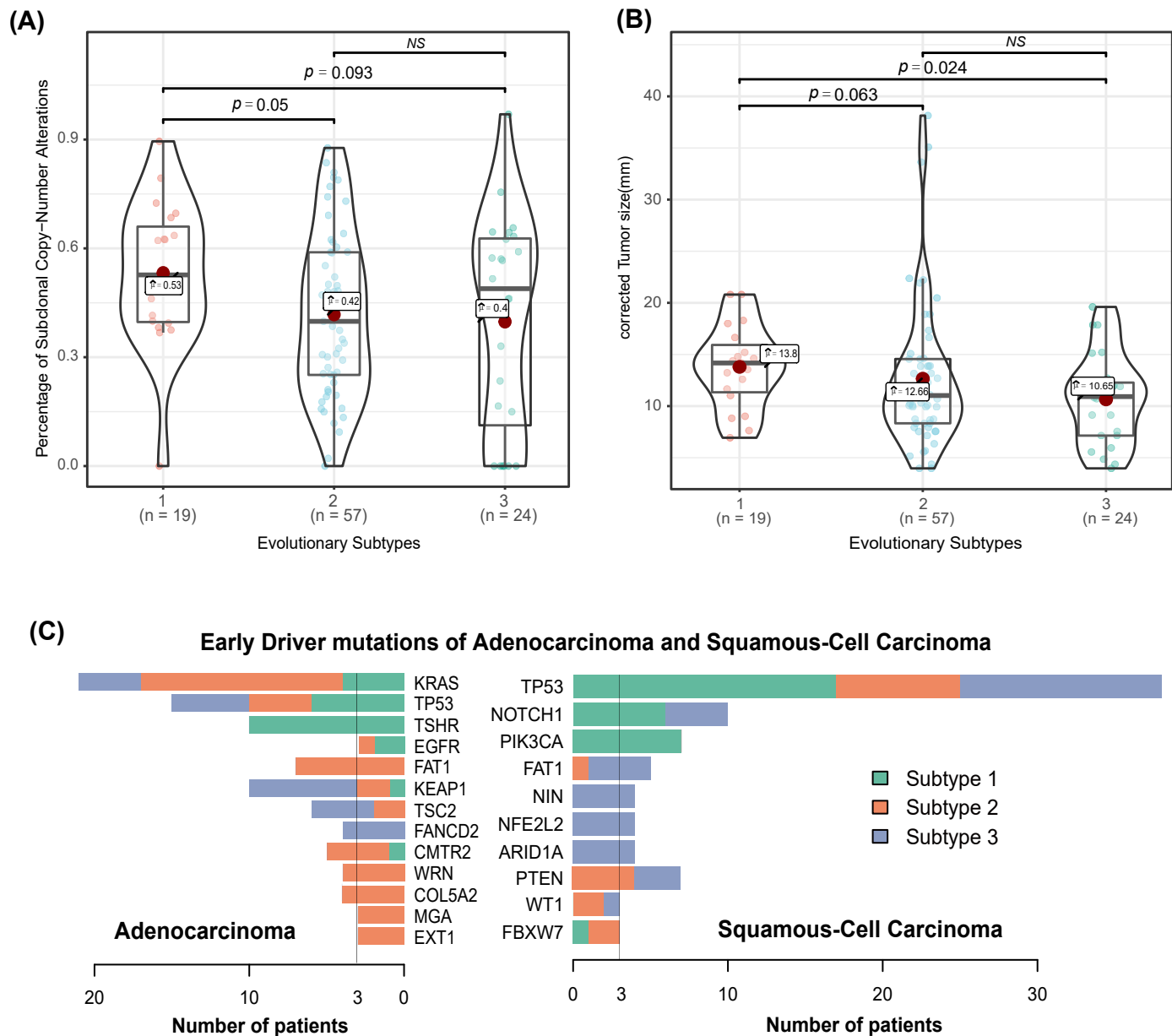

Figure S5. The correlations between the copy number ITH and evolutionary subtypes.

Supplement: Supplementary file 5 — Fig S5 [file CAM4-9-9485-s005.pdf]

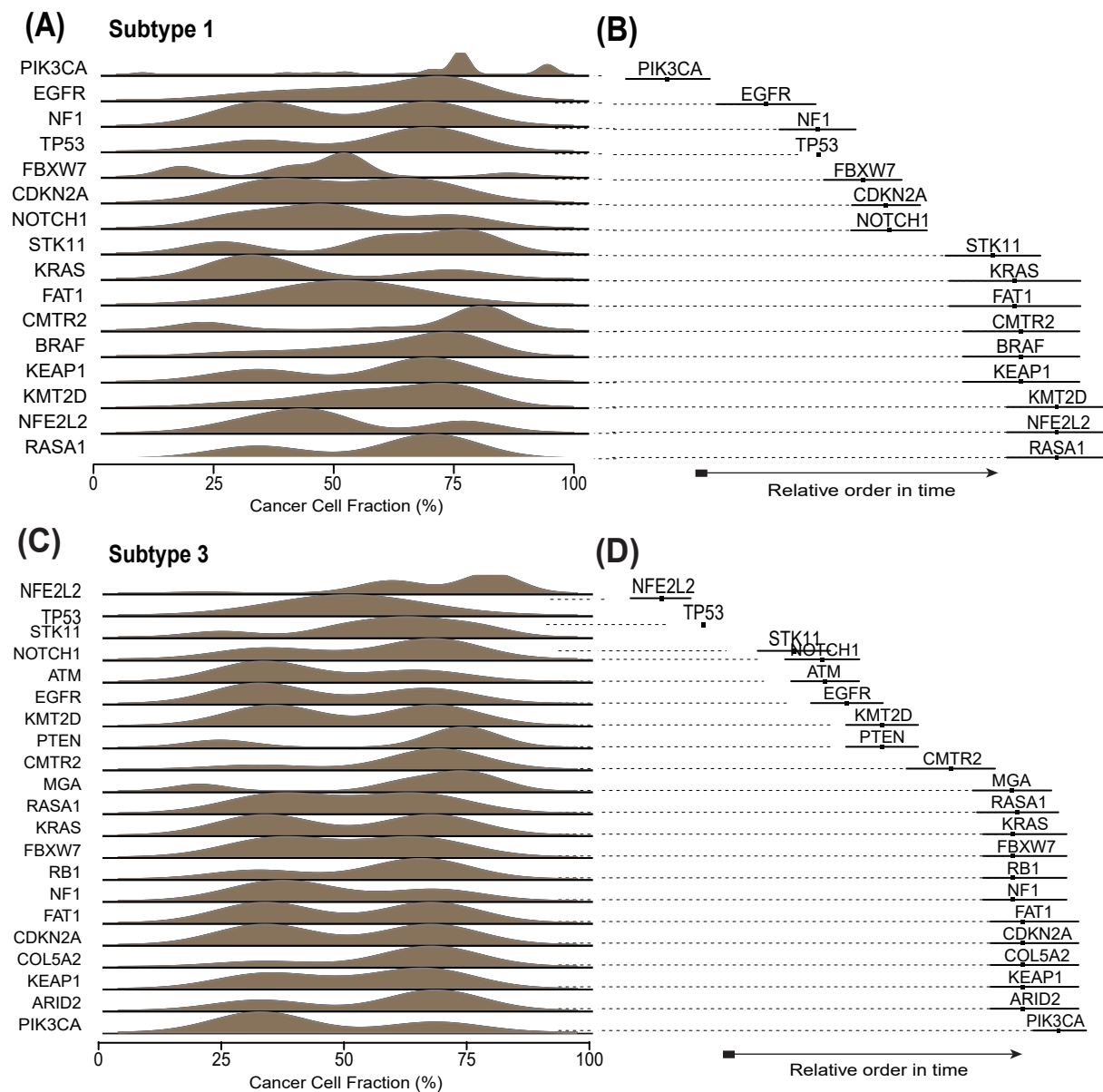

Figure S6. The temporal order of driver mutations across subtype 1 and 3.

Supplement: Supplementary file 6 — Fig S6 [file CAM4-9-9485-s006.pdf]

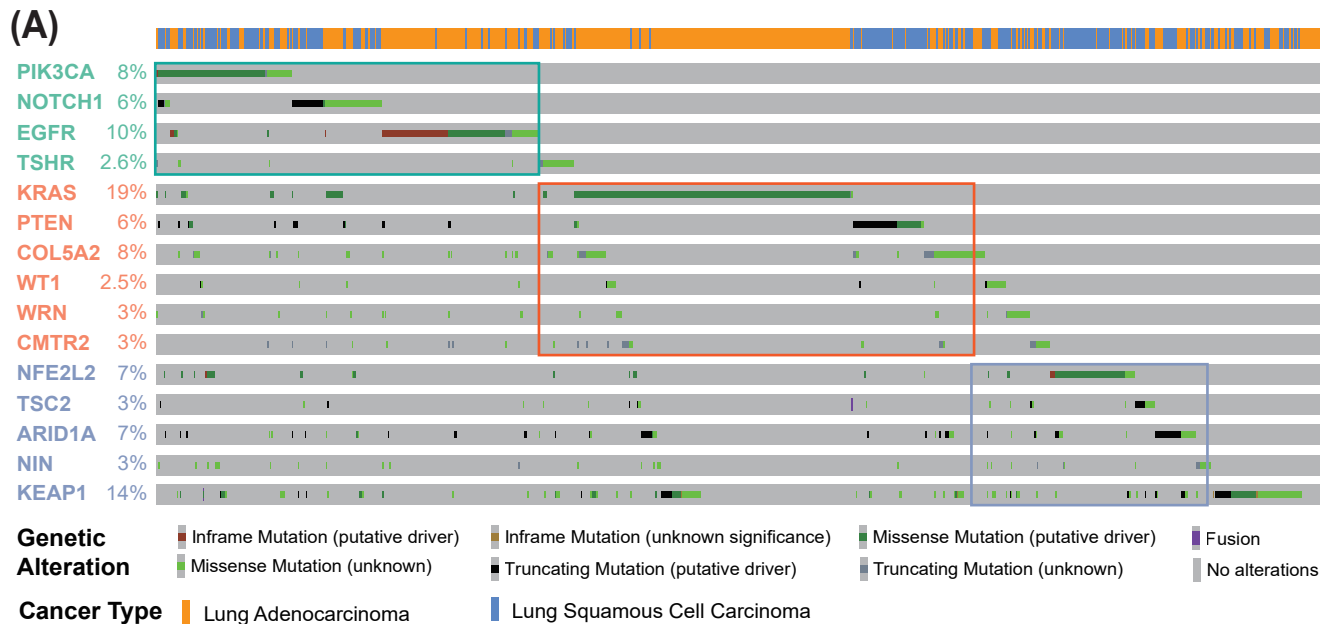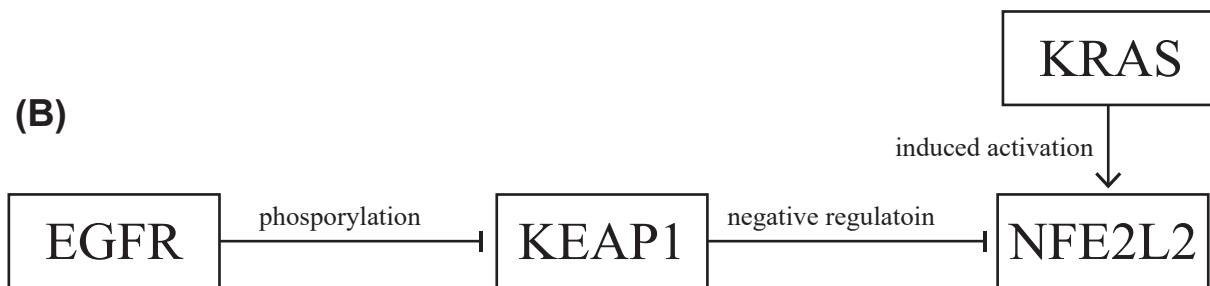

Figure S7. Genomic characteristics of the EDF genes.

Supplement: Supplementary file 7 — Fig S7 [file CAM4-9-9485-s007.pdf]
