# Supplementary material for: Revealing the subtyping of non‐small cell lung cancer based on genomic evolutionary patterns by multi‐region sequencing
Source: Cancer Med. 2020 Oct 20;9(24):9485–98. doi: 10.1002/cam4.3541 (PMC7774747; doi:10.1002/cam4.3541)
Supplement: Supplementary file 3 — Fig S3 [file CAM4-9-9485-s003.pdf]

Phylogenetic trees for TRACERx 100 Cohort

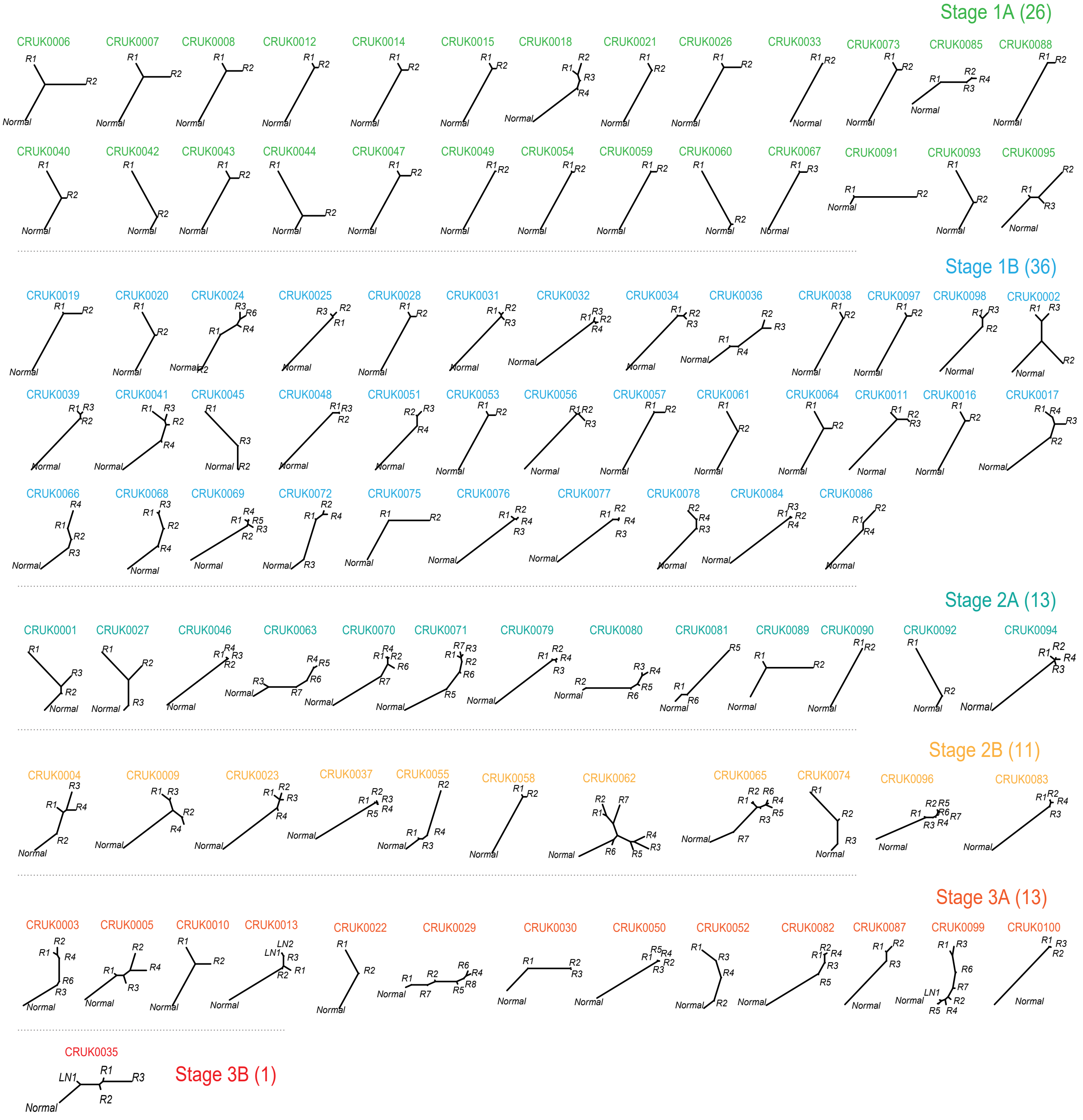

Figure S3. Phylogenetic trees for 100 NSCLC patients.
